# Fewer Injections and Lower Drug Costs With Long‐Acting GnRH Depot Formulations: A Nationwide Analysis of 2.3 Million Patient‐Years in Japan

**DOI:** 10.1002/cnr2.70688

**Published:** 2026-09-11

**Authors:** Takashi Kawahara, Akihito Hashizume, Masanobu Yamazaki, Daiji Takamoto, Teppei Takeshima, Hiroji Uemura, Kazuhide Makiyama, Jun‐ichi Teranishi

**Affiliations:** ^1^ Department of Urology Yokohama City University Medical Center Yokohama Japan; ^2^ Department of Renal Transplantation Yokohama City University Medical Center Yokohama Japan; ^3^ Department of Urology Yokohama City University, Graduate School of Medicine Yokohama Japan

**Keywords:** androgen deprivation therapy, depot formulation, drug cost, gonadotropin‐releasing hormone, prostate cancer

## Abstract

**Background:**

Androgen deprivation therapy (ADT) is the base of treatment for prostate cancer, and gonadotropin‐releasing hormone (GnRH) drugs are given as depot injections. Long‐acting formulations may reduce the number of injections and the drug cost, but this impact has not been quantified in real practice.

**Methods:**

We used the National Database Open Data of Japan from fiscal year (FY) 2017 to FY2024, and studied leuprorelin, goserelin, and degarelix in men aged 40 years or older. Patient‐years were estimated by weighting the dispensed quantity by the period that each formulation covers. Descriptive statistics (patient numbers, drug, and formulation shares) were based on all products, whereas the analyses of the number of administrations and drug cost were restricted to originator products. Shapley value decomposition was used to divide the change in injections and in drug cost into factors.

**Results:**

In total, 2 276 025 patient‐years were analyzed. The share of each drug did not change greatly, but a clear shift to long‐acting formulations was seen. The 6‐month formulation of leuprorelin exceeded the 3‐month formulation in FY2021 and was used more often in older patients. The number of patients increased by 12.9% (from 269 328 to 304 181 patient‐years; +10.0% for originator products, used for the administration and cost analyses), but injections decreased by 21.5%, from 1 275 081 to 1 000 324. Injections per patient per year decreased from 4.97 to 3.55. The shift to long‐acting formulations was the main factor for this decrease (−383 932 injections), and it also reduced the drug cost by 7.7 billion JPY per year.

**Conclusions:**

The shift to long‐acting GnRH formulations was associated with a reduction in injections of about one in five, even though the number of patients increased and was estimated to account for part of the reduction in drug cost. Because the oncological effect is similar among GnRH drugs, a further shift to long‐acting formulations may lower the burden on patients and on health care.

## 1 Introduction

Treatment for prostate cancer has advanced in recent years. Androgen receptor signaling inhibitors and docetaxel improve survival when they are added to androgen deprivation therapy (ADT) in metastatic hormone‐sensitive prostate cancer, and treatment options have increased [1, 2]. However, all of these combination therapies are based on ADT. Therefore, hormonal therapy is still the base of treatment for prostate cancer. In Japan, three injectable gonadotropin‐releasing hormone (GnRH) drugs are used. Two of them are agonists, leuprorelin and goserelin, and one is an antagonist, degarelix. Each drug has formulations with different durations of action, such as 1‐month, 3‐month, and 6‐month depots. The available formulations differ among the drugs [3, 4].

Several factors affect the choice of a GnRH drug. These include patient preference [5], the impact on cardiovascular events [6, 7], and the evidence for combination with radiotherapy [8]. However, a recent meta‐analysis showed no clear difference in oncological outcomes between GnRH antagonists and agonists [9]. Therefore, the oncological effect is generally considered to be similar among these drugs.

In this situation, the shift to long‐acting formulations is important. It affects not only drug cost, but also the number of injections that a patient receives. A smaller number of injections may reduce the cost of medical care, the burden of hospital visits for patients, and the workload of health‐care staff who perform the injections [3, 4]. In Japanese patients with prostate cancer, formulations with a longer interval were preferred [5]. However, few studies have focused on the drug cost and the number of injections of GnRH drugs. In Japan, the National Database (NDB) Open Data has been used to describe drug use in several fields [10, 11]. To our knowledge, no study has quantified the change in the choice of formulation duration of GnRH drugs, or its impact on drug cost and the number of injections. Therefore, we used the NDB Open Data, which covers almost all insurance claims in Japan. The aim of this study was to describe the use of GnRH drugs for prostate cancer, to show the change in the duration of action of the formulations that were used, and to quantify the impact of this change on drug cost and the number of injections.

## 2 Materials and Methods

This was a descriptive study that used the National Database (NDB) Open Data, which is published by the Ministry of Health, Labour and Welfare of Japan. The NDB contains almost all health insurance claims in Japan. In the Open Data, the dispensed quantity of each drug is aggregated by the type of care (outpatient care in the hospital, outpatient care with an outside pharmacy, and inpatient care) and by sex and age group. We used the data on injectable drugs from fiscal year (FY) 2017 to FY2024, and we combined the three types of care for each fiscal year. We studied the GnRH drugs that are used for androgen deprivation therapy (ADT) in prostate cancer: leuprorelin acetate (including generic products), goserelin acetate, and degarelix acetate. To exclude the indications for women (premenopausal breast cancer, endometriosis, and uterine fibroids), we included only men. To exclude central precocious puberty in boys, we also limited the analysis to men aged 40 years or older. In addition, we excluded leuprorelin 1.88 mg and goserelin 1.8 mg, because these formulations are not indicated for prostate cancer. The sex‐ and age‐specific data for FY2022 in the combined dataset were inconsistent, so we replaced this year with the data from the original NDB file. This study was conducted in accordance with the Declaration of Helsinki and was approved by the Institutional Review Board of Yokohama City University Medical Center, Yokohama, Japan (approval number: F260700015). This study used only publicly available, anonymized, and aggregated open data (NDB Open Data), and did not handle any individual patient data or identifiable personal information; therefore, the requirement for informed consent was waived.

The NDB Open Data provides the dispensed quantity (the number of vials or kits) and not the number of unique patients. Because the NDB Open Data is aggregated and cannot track individuals over time, patient‐years represent estimated treatment volume derived from dispensed quantities and not counts of unique individuals. Therefore, we estimated patient‐years by weighting the dispensed quantity by the period that each formulation covers. We multiplied the 1‐month formulation by 1/12, the 3‐month formulation by 1/4, and the 6‐month formulation by 1/2. For degarelix, we considered the composition of the vials. The maintenance dose of 80 mg (one vial per month) was multiplied by 1/12, the loading dose of 120 mg (two vials for one administration) by 1/24, and the 3‐month formulation of 240 mg (two vials for one administration every 84 days) by 1/8. We calculated the number of administrations in the same way, and we counted the loading dose and the 3‐month formulation of degarelix as one administration because two vials are used at one visit. The drug cost was calculated by multiplying the dispensed quantity of each product by the reimbursement price of that fiscal year. The analysis of drug cost was limited to originator products.

We used Shapley value decomposition to divide the change in drug cost and in the number of administrations between FY2017 and FY2024 into factors. For drug cost, we used three factors: the number of patients, the revision of drug prices, and the shift in formulation duration (from 1‐month to 3‐month and 6‐month). For the number of administrations, we used two factors: the number of patients and the shift in formulation duration. The Shapley value of a factor is defined as its mean marginal contribution over all possible orders in which the factors are introduced: for a value function v and factor *i*, phi_*i* = sum over subsets S not containing *i* of [|S|! (*n*−|S|−1)!/*n*!] × [*v*(S union [9]) − *v*(S)], where *n* is the number of factors [12]. Because the three GnRH agents differ in price and in the depot durations available, the effect of a change in agent selection cannot be fully separated from the change in formulation duration; we therefore combined agent selection and formulation duration into a single “formulation shift” factor for the main analysis, and confirmed in a preliminary four‐factor model that the independent contribution of agent selection was negligible (less than 0.1 billion JPY). The Shapley value is the mean of the marginal contribution of each factor over all possible orders of the factors. Therefore, it does not depend on the order, and the sum of the contributions is exactly equal to the observed change (residual = 0). The cost reduction by the shift to long‐acting formulations depends on the conditions under which the other factors (the number of patients and drug prices) are evaluated. Therefore, we performed a sensitivity analysis. We calculated the reduction under FY2017 conditions, under FY2024 conditions, when only drug prices were set to FY2024, and when only the number of patients was set to FY2024. We used Python (pandas, NumPy, openpyxl, matplotlib) for the analysis. The authors used Claude (Anthropic, San Francisco, CA, USA) to assist with writing the code used to generate the figures, and with editing and proofreading the English text. The authors reviewed and verified all output and take full responsibility for the content of this manuscript.

## 3 Results

### 3.1 Patients, Drugs, and Formulations

**FIGURE 1:**
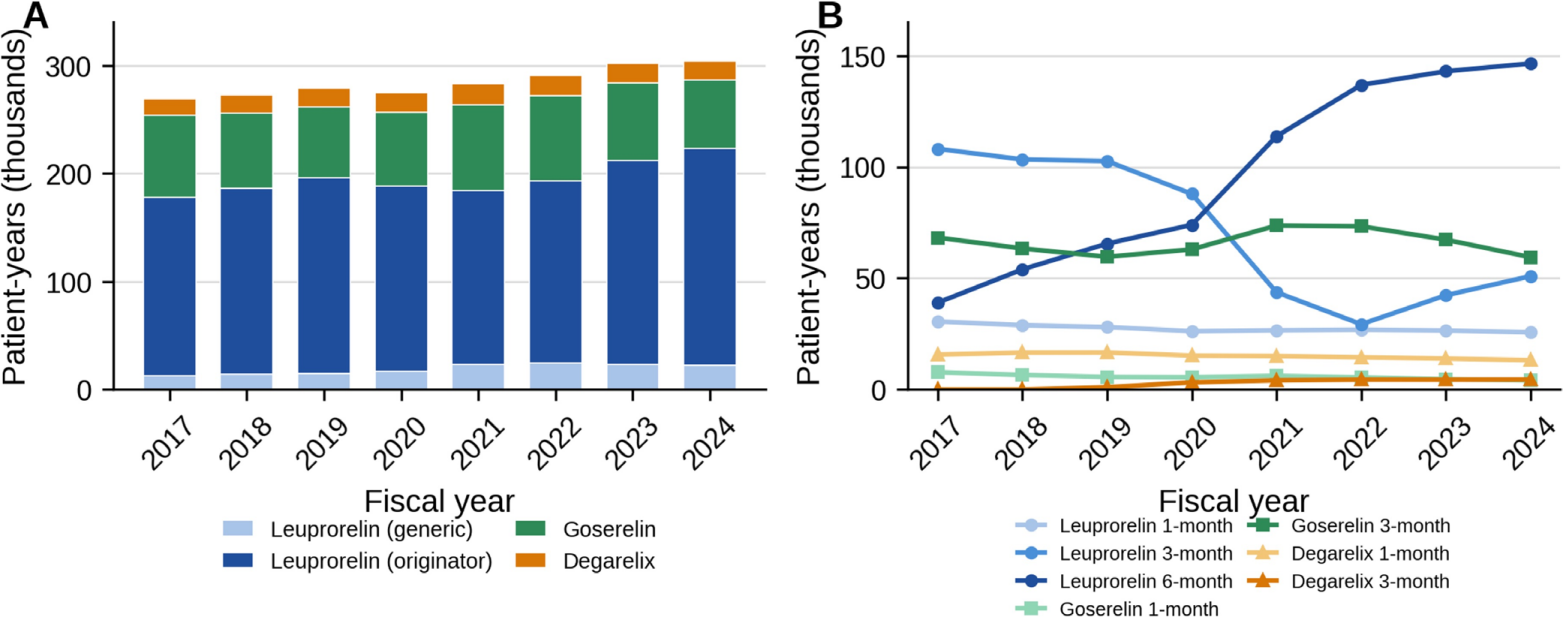
Trends in the number of patients receiving injectable ADT. (a) Number of patients by drug. Leuprorelin is shown separately for originator and generic products. (b) Number of patients by drug and formulation. Patient‐years were calculated by multiplying the dispensed units by the period that each formulation covers (1‐month ×1/12; 3‐month ×1/4; 6‐month ×1/2). Men aged 40 years or older.

**FIGURE 2:**
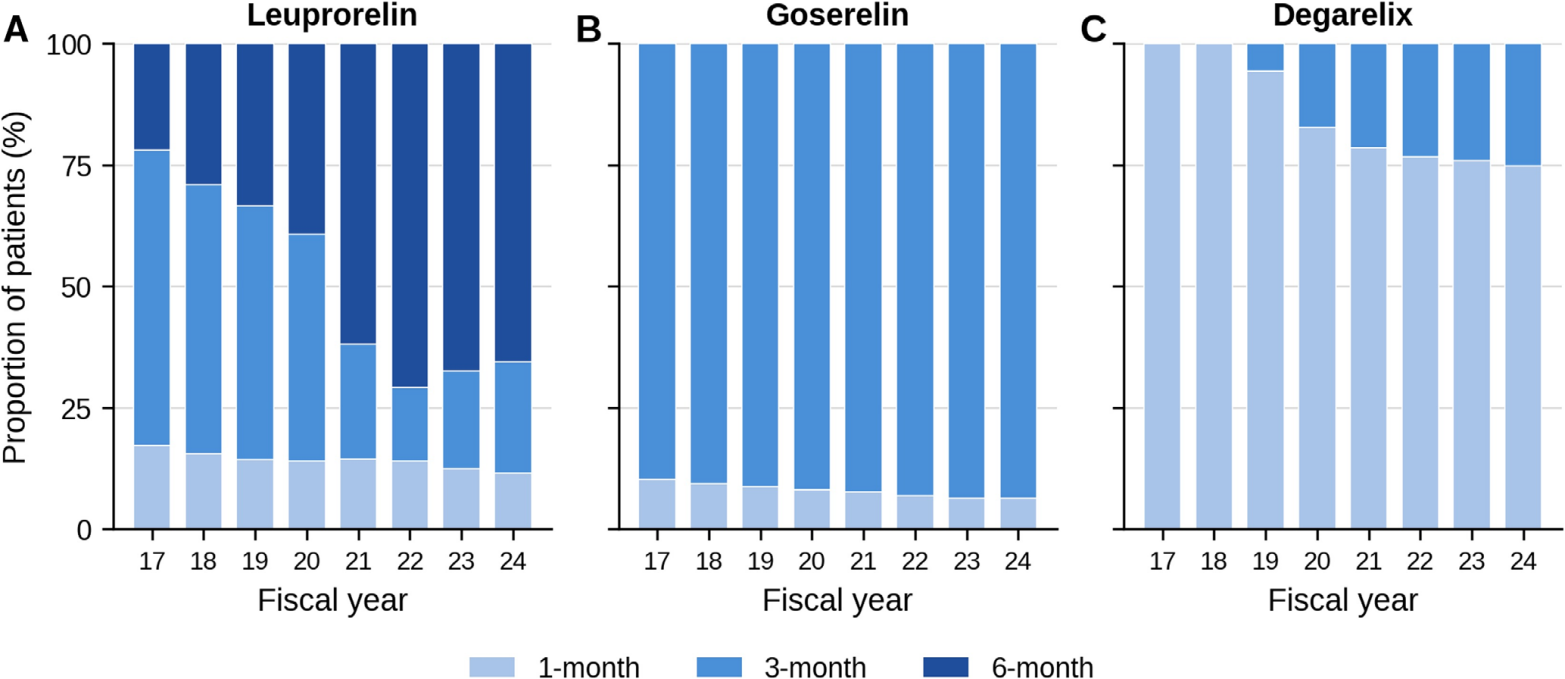
Distribution of depot formulation duration within each agent. (a) Leuprorelin, (b) goserelin, and (c) degarelix. Bars show the proportion of patients who received the 1‐month, 3‐month, or 6‐month formulation. Only leuprorelin has a 6‐month formulation. The 3‐month formulation of degarelix was introduced in FY2019.

**FIGURE 3:**
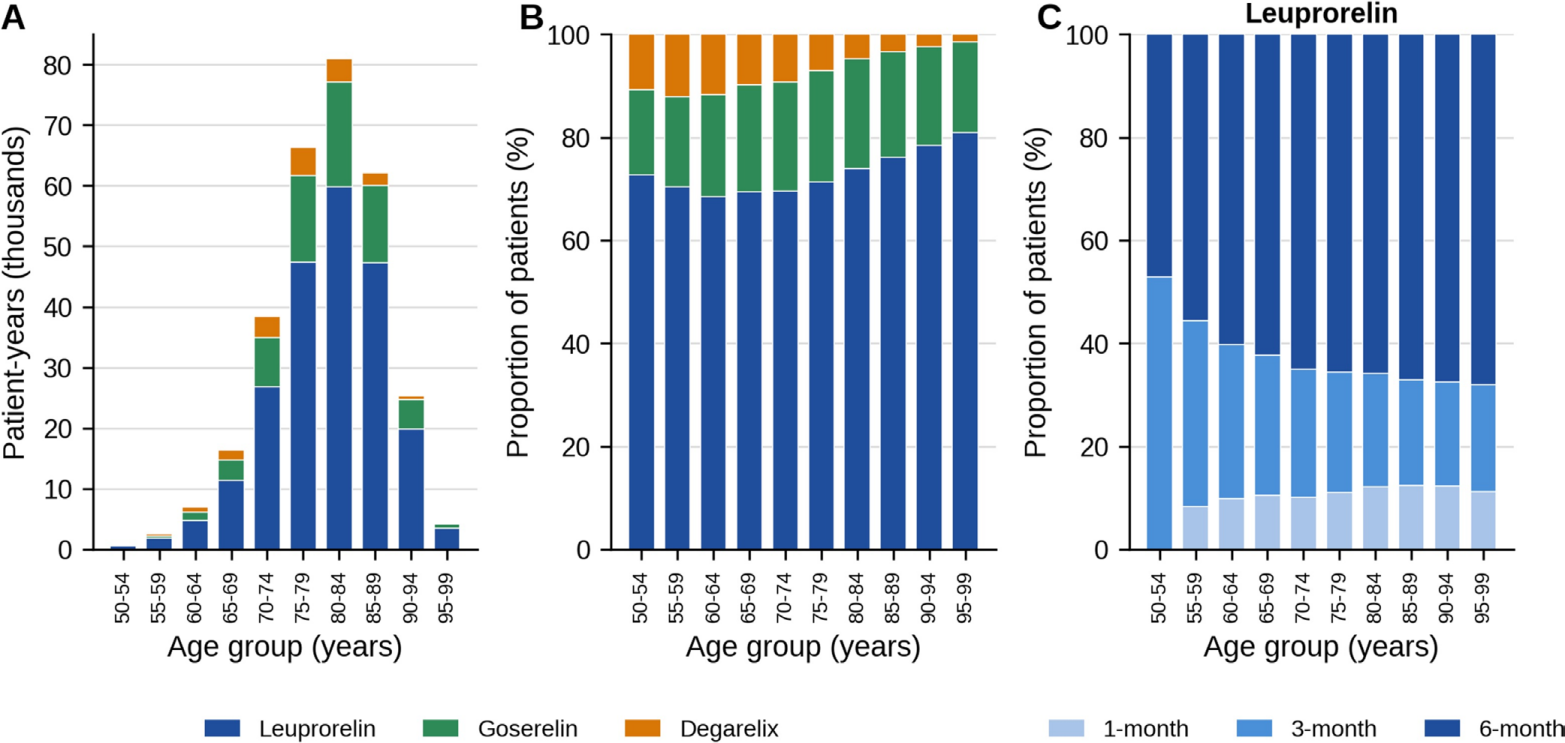
Age‐stratified distribution of patients and treatment patterns, FY2024. (a) Number of patients by age group and drug, (b) share of each drug by age group, and (c) share of each formulation by age group, among patients who received leuprorelin.

We used the NDB Open Data from fiscal year (FY) 2017 to FY2024. We studied injectable GnRH agonists and antagonists that are used for prostate cancer. To study prescriptions for prostate cancer, we excluded women. We also limited the analysis to men aged 40 years or older, because leuprorelin is also used for central precocious puberty in boys (Table 1). In total, 2 276 025 patient‐years were included. The mean age was 79.8 years, and 46.7% of patients were aged 80–89 years. Three drugs were used: leuprorelin, goserelin, and degarelix. Generic products were available only for the 1‐month formulation of leuprorelin (3.75 mg). No generic product was available for the 3‐month formulation (11.25 mg) or the 6‐month formulation (22.5 mg), even in FY2024. Considering all products (including generics), the number of patients increased by 12.9%, from 269 328 patient‐years in FY2017 to 304 181 patient‐years in FY2024. The share of each drug did not change greatly. However, the share of leuprorelin increased from 66.0% to 73.3%, and the share of goserelin decreased from 28.2% to 20.9%. The share of degarelix did not change (5.8%) (Figure 1a). When we looked at the formulations, the change was clear. The 3‐month formulation of leuprorelin decreased from 108 112 patient‐years in FY2017. The 6‐month formulation increased from 39 005 to 146 478 patient‐years. In FY2021, the 6‐month formulation exceeded the 3‐month formulation in the number of patients (Figure 1b). We then looked at each drug separately. For leuprorelin, the share of the 6‐month formulation increased from 22.0% in FY2017, but it reached a plateau after FY2022 (70.9%) and was 65.7% in FY2024. In the same period, the share of the 3‐month formulation increased again, from 15.2% to 22.8% (Figure 2a). For goserelin, the 3‐month formulation was dominant from FY2017 (89.8%), and it did not change much (93.6% in FY2024) (Figure 2b). For degarelix, the 3‐month formulation was introduced in FY2019, and it increased slowly to 25.2% in FY2024 (Figure 2c). The number of patients had a peak at 80–84 years (80 942 patient‐years in FY2024) (Figure 3a). The share of goserelin was almost constant across age groups (17%–21%). The share of degarelix was higher in younger patients (10.8% at 50–54 years) and decreased with age (1.5% at 95–99 years). In contrast, leuprorelin was used more often in older patients (from 72.6% to 80.9%) (Figure 3b). When we looked only at patients who received leuprorelin, the share of the 6‐month formulation increased with age (47% at 50–54 years and 68% at 95–99 years), and it replaced the 3‐month formulation. This age gradient was not seen in FY2017, when the share was 22%–23% in all age groups. Therefore, this gradient appeared in recent years (Figure 3c).

**TABLE 1:** Characteristics of patients receiving injectable ADT.

Characteristics	Total (FY2017–FY2024)	FY2017	FY2024	Change
Patients (patient‐years)	2 276 025	269 328	304 181	+12.9%
Age, mean (years)	79.8	79.4	80.1	+0.7
Age group, *n* (%)				
60–69	190 283 (8.4)	26 325 (9.8)	23 260 (7.6)	−2.2
70–79	809 546 (35.6)	96 463 (35.8)	104 830 (34.5)	−1.3
80–89	1 062 589 (46.7)	126 500 (47.0)	143 048 (47.0)	0.0
≥ 90	190 403 (8.4)	17 844 (6.6)	29 567 (9.7)	+3.1
Agent, *n* (%)				
Leuprorelin	1 560 240 (68.6)	177 679 (66.0)	223 100 (73.3)	+7.3
Goserelin	573 644 (25.2)	76 000 (28.2)	63 481 (20.9)	−7.3
Degarelix	142 141 (6.2)	15 649 (5.8)	17 599 (5.8)	0.0
Depot duration, *n* (%)				
1‐month	385 801 (17.0)	53 978 (20.0)	42 924 (14.1)	−5.9
3‐month	1 117 637 (49.1)	176 344 (65.5)	114 779 (37.7)	−27.8
6‐month	772 587 (33.9)	39 005 (14.5)	146 478 (48.2)	+33.7
Burden and cost (originator only)				
Administrations, *n*	8 828 394	1 275 081	1 000 324	−21.5%
Administrations per patient‐year	4.16	4.97	3.55	−28.6%
Total drug cost (billion JPY)	443.8	70.4	42.1	−40.2%
Drug cost per patient‐year (JPY)	208 906	274 708	149 277	−45.7%

*Note:* The total column shows the cumulative value across the eight fiscal years. Patient‐years were estimated by weighting dispensed units by the treatment duration covered by each depot formulation. Men aged ≥ 40 years. The change column shows percentage‐point differences for proportions and relative changes elsewhere.

### 3.2 Number of Administrations

**FIGURE 4:**
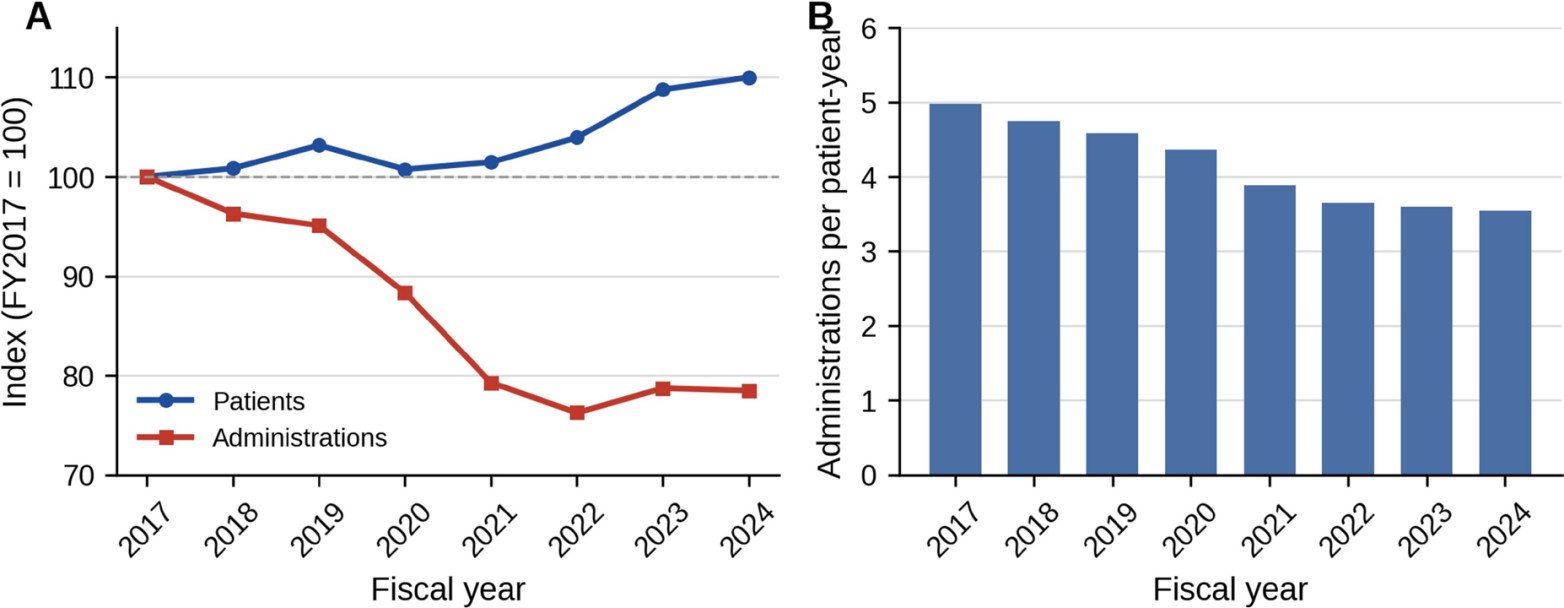
Trends in the number of drug administrations. (a) Number of patients and number of administrations (FY2017 = 100) and (b) number of administrations per patient per year. For degarelix, the loading dose (120 mg × 2 vials) and the 3‐month formulation (240 mg × 2 vials) were counted as one administration. Originator products only.

We next looked at this trend from the view of the number of injections. In the originator‐only population used for this analysis, the number of patients increased slightly (+10.0%; +12.9% for all products including generics). However, the total number of administrations decreased by 21.5%, from 1 275 081 to 1 000 324 (Figure 4a). As a result, the number of administrations per patient per year decreased by 28.7%, from 4.97 in FY2017 to 3.55 in FY2024 (Figure 4b).

### 3.3 Drug Cost and Decomposition

**FIGURE 5:**
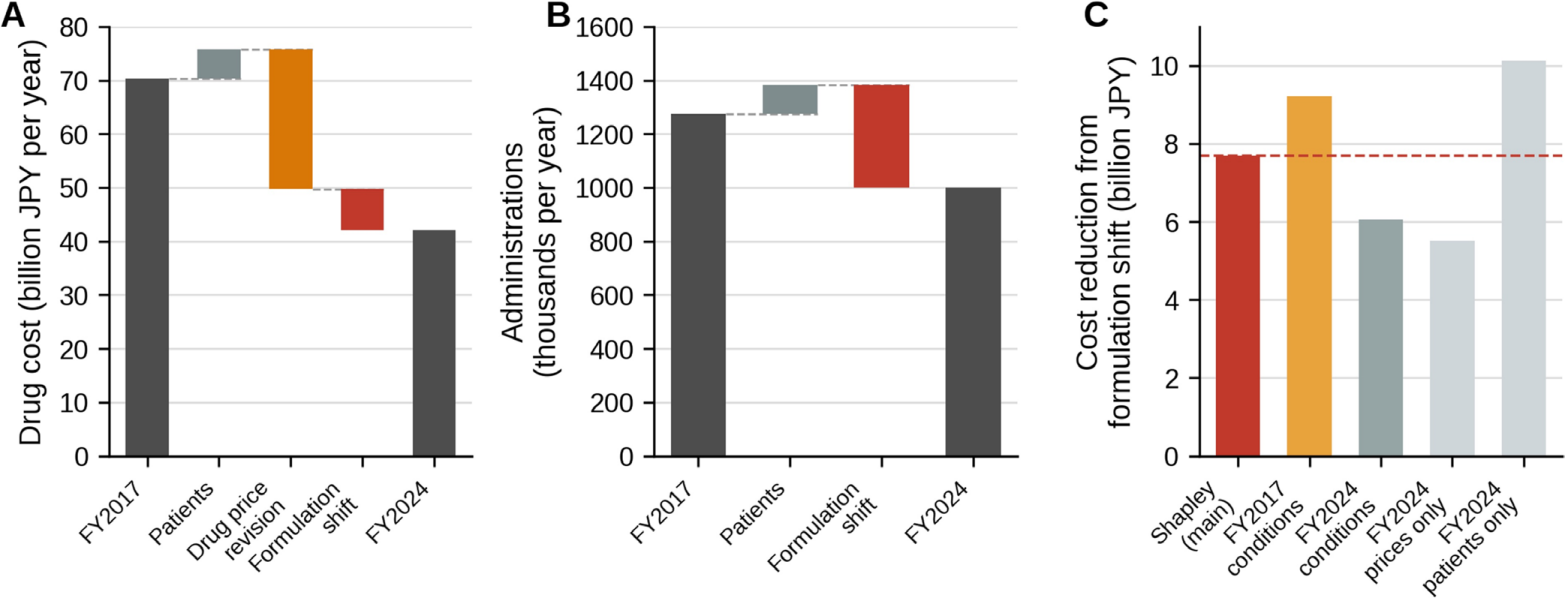
Shapley decomposition of changes between FY2017 and FY2024. (a) Change in drug cost, divided into three factors, (b) change in the number of administrations, divided into two factors, and (c) cost reduction by the shift to long‐acting formulations, under different conditions of calculation. The factors sum to the total change (residual = 0). Originator products only.

The annual drug cost per patient differed greatly among the formulations. At FY2017 prices, it was 453 324 JPY for the 1‐month formulation of leuprorelin, 267 564 JPY for the 3‐month formulation, and 204 828 JPY for the 6‐month formulation (Figure S1). We then looked at the change in drug cost (originator products only). The drug cost decreased by 28.3 billion JPY, from 70.4 billion JPY in FY2017 to 42.1 billion JPY in FY2024. Using Shapley value decomposition, this change was divided into three factors: the increase in the number of patients (+5.4 billion JPY), the revision of drug prices (−26.0 billion JPY), and the shift to long‐acting formulations (−7.7 billion JPY). The sum of the three factors was equal to the total change (residual = 0) (Figure 5a). We used the same method for the total number of administrations in Japan (−274 757). The increase in the number of patients raised the number of administrations by 109 176. The shift to long‐acting formulations reduced it by 383 932. Therefore, the shift to long‐acting formulations was the main factor (Figure 5b). The cost reduction by the shift to long‐acting formulations depended on the conditions used for the calculation. It was 7.7 billion JPY by the Shapley value, 9.2 billion JPY under FY2017 conditions, 6.1 billion JPY under FY2024 conditions, 5.5 billion JPY when only drug prices were set to FY2024, and 10.1 billion JPY when only the number of patients was set to FY2024 (Figure 5c).

## 4 Discussion

In this study, we showed the change in the use of GnRH drugs for prostate cancer in Japan from FY2017 to FY2024. The share of each drug did not change greatly. The share of leuprorelin increased from 66.0% to 73.3%, the share of goserelin decreased from 28.2% to 20.9%, and the share of degarelix did not change (5.8%). In contrast, a clear shift from short‐acting to long‐acting formulations was seen in all three drugs. For leuprorelin, the 6‐month formulation replaced the 3‐month formulation, and it exceeded the 3‐month formulation in FY2021. The 6‐month formulation of leuprorelin was used more often in older patients (47% at 50–54 years and 68% at 95–99 years). This shift to long‐acting formulations was associated with a decrease in drug cost, estimated at 7.7 billion JPY per year (5.5–10.1 billion JPY, depending on the conditions of calculation), and the number of injections decreased by 383 932 per year. The NDB Open Data covers almost all insurance claims in Japan. Therefore, we consider that our study captured this shift in the number of injections in real practice.

A smaller number of injections reduces the burden of hospital visits, and it may improve the quality of life of patients. In our previous study, we analyzed 851 hormonal injections and evaluated the time from prescription to the end of the injection, and also the physical and mental burden on patients [5]. Leuprorelin 22.5 mg (the 6‐month formulation) reduced the time in the hospital the most, and the burden on patients was the smallest. In contrast, degarelix needed a longer time because the drug must be reconstituted and the injection site must be cooled to prevent skin reactions [5]. For goserelin, a large‐bore needle is needed because of the depot implant, and this may also be a burden. These findings support a further shift to long‐acting formulations. However, our study showed only the number of injections. We did not measure how much the burden on patients was actually reduced. We did not measure the quality of life, the time and cost of hospital visits, or the burden on caregivers. The change from 4.97 to 3.55 injections per patient per year should be evaluated in future prospective studies. The effect of fewer injections on patient satisfaction, the number of hospital visits, and caregiver burden was not measured in this study and should be evaluated prospectively.

At present, most patients who receive goserelin already use the 3‐month formulation (93.6% in FY2024). In contrast, the 3‐month formulation of degarelix is increasing every year, but it was still used in only 25.2% of patients in FY2024, and most patients still use the 1‐month formulation. The 3‐month formulation of degarelix (a maintenance dose of 480 mg, given as 240 mg × 2 vials every 84 days) was developed in Japan, and Phase II [13] and Phase III [14] studies were performed in Japanese patients. In the United States and Europe, only the 1‐month regimen of degarelix (240 mg loading dose and 80 mg every 28 days) is approved. Therefore, the 3‐month formulation is available only in Japan, and this is the first report that shows how it is used in real practice.

A further way to reduce the burden of injections is the oral GnRH antagonist relugolix, which avoids injections entirely and achieves rapid testosterone suppression [15]. Relugolix is not included in the present analysis because it is an oral drug and is not captured in the injectable data used here. The uptake of oral agents may further change the pattern of ADT in the future, and should be monitored [15].

We also need to consider the supply problem of leuprorelin during the study period. Because of re‐validation at the manufacturing plant, the supply was limited from June 2020. The 3‐month formulation (11.25 mg) was out of stock, and the 1‐month and 6‐month formulations were also limited. The lifting of this restriction was announced on 28 July 2022, and shipments returned to normal in September 2022 [16]. Our data agree with this problem. The 3‐month formulation of leuprorelin decreased in FY2021 and FY2022 (from 43 686 to 29 304 patient‐years), and it recovered in FY2023 and FY2024 after the supply became normal (from 42 346 to 50 925 patient‐years). In the same period, the share of generic products, which are available only for the 1‐month formulation, reached a peak in FY2022 (8.4%) and then decreased to 7.3%. This suggests that generic products were used as an alternative during the shortage. The share of the 6‐month formulation also reached a plateau after FY2022 (70.9%), and this may be related to the limited supply. Therefore, part of the shift to long‐acting formulations may reflect the supply of drugs, and not only the preference of doctors and patients. However, the 6‐month formulation kept the largest share in FY2023 and FY2024, when the supply was normal. This suggests that the shift is not a temporary substitution, but a practice pattern that has become established. To test whether the observed shift merely reflected this shortage, we repeated the decomposition of the number of administrations for periods less affected by the shortage. The formulation shift reduced administrations in the pre‐shortage period (FY2017–FY2020, −157 487) and also after the supply had normalized (FY2022–FY2024, −27 717; FY2023–FY2024, −14 376). Thus, the shift to long‐acting formulations reduced the number of injections in every sub‐period, and was not explained by the shortage alone.

This study has several limitations. First, we used aggregated data on the total number of prescriptions, and we could not follow each patient. We could not identify patients who received a GnRH antagonist first to avoid a flare‐up and then changed to a GnRH agonist, or patients who changed to another drug because of adverse events. Future studies with patient‐level data are needed to show how each patient changes drugs. Second, this study is a decomposition of observational data, and it is not a causal analysis. The choice of a long‐acting formulation may be confounded by the background of the patient, such as the stage of disease, the ability to visit the hospital, the general condition, and the prognosis. In fact, the 6‐month formulation was used more often in older patients, and degarelix was used more often in younger patients. Therefore, the choice of formulation is biased.

Third, in the NDB Open Data, counts of less than 400 are masked. Therefore, the use of drugs in small numbers may be underestimated. In addition, the drug cost was calculated from the official reimbursement price, and it may differ from the actual purchase price of each hospital.

## 5 Conclusion

From FY2017 to FY2024 in Japan, the number of patients who received GnRH drugs for prostate cancer increased from 269 328 to 304 181 patient‐years. The share of each drug did not change greatly, but a clear shift to long‐acting formulations was seen, and the 6‐month formulation of leuprorelin exceeded the 3‐month formulation in FY2021. During this period, the number of patients increased by 12.9% for all products (10.0% for originator products, on which the administration and cost analyses were based), but the total number of injections decreased by 21.5% (from 1 275 081 to 1 000 324), and the number of injections per patient per year decreased from 4.97 to 3.55. In the decomposition, the shift to long‐acting formulations was the factor most strongly associated with the change in the number of injections (−383 932). For the change in drug cost (−28.3 billion JPY), the shift to long‐acting formulations was estimated to contribute −7.7 billion JPY.

## Author Contributions

**Takashi Kawahara:** data curation, conceptualization, methodology, investigation, writing – original draft, funding acquisition. **Akihito Hashizume:** data curation. **Masanobu Yamazaki:** data curation. **Daiji Takamoto:** investigation. **Teppei Takeshima:** supervision. **Hiroji Uemura:** supervision. **Kazuhide Makiyama:** supervision. **Jun‐ichi Teranishi:** supervision.

## Funding

The authors have nothing to report.

## Conflicts of Interest

The authors declare no conflicts of interest.

## Supporting information


**Data S1:** Supporting Information.

**Figure S1:** Annual drug cost per patient‐year. Bars show the cost to treat one patient for 1 year with each formulation, at FY2017 and FY2024 prices. Generic products were excluded.

## Data Availability

The raw data to create tables and figures are available as a Supplementary file.
